# Correlation between inflammatory factors, autophagy protein levels, and infection in granulation tissue of diabetic foot ulcer

**DOI:** 10.1002/iid3.1233

**Published:** 2024-04-05

**Authors:** Lijuan Deng, Gang Wang, Shang Ju

**Affiliations:** ^1^ Department of Peripheral Vascular, Dongzhimen Hospital Beijing University of Chinese Medicine Beijing China

**Keywords:** autophagic proteins, correlation, diabetic foot ulcer, granulation tissue, inflammatory factors, infection

## Abstract

**Objective:**

To observe the expression of inflammatory factors and autophagy‐related proteins in granulation tissue of diabetic foot ulcer (DFU) patients and analyze their relationship with infection.

**Methods:**

This is a retrospective cohort study. One hundred and fifty‐two patients with DFU in our hospital from July 2020 to March 2022 were selected as the DFU group, including 98 cases in infection stage group and 54 cases in infection control group. The patients were further graded as the mild (51 cases), the moderate (65 cases), and the severe infection group (36 cases) according to the Wagner grading criteria. Sixty‐seven patients with foot burns during the same period were selected as the control group. The distribution of pathogenic bacteria on the ulcer surface was examined using fully automated bacterial analyzer. The expression of inflammatory factors (procalcitonin [PCT], tumor necrosis factor‐α [TNF‐α], and interleukin‐6 [IL‐6]) was valued by real‐time fluorescence quantitative PCR (qRT‐PCR). Protein expression was measured by immunohistochemistry (IHC). The correlation was analyzed by Pearson.

**Results:**

The surface infection of DFU patients was mostly induced by gram‐negative and gram‐positive bacteria, with *Pseudomonas aeruginosa* predominating among the Gram‐negative bacteria and *Staphylococcus aureus* among the gram‐positive bacteria. The infection stage group had higher content of PCT, TNF‐α, and IL‐6 and lower content of Beclin‐1 and LC3 than the infection control group (*p* < .001). The levels of PCT, TNF‐α, and IL‐6 in the DFU patients with cardiovascular events were higher than those in the nonoccurrence group (*p* < .001). Glycated hemoglobin in patients with DFU was positively correlated with PCT, TNF‐α, and IL‐6 levels (*p* < .05), and negatively correlated with Beclin‐1 and LC3 levels (*p* < .001).

**Conclusion:**

*P. aeruginosa* and *S. aureus* were predominant bacterial in DFU infections. Inflammatory factor and autophagy protein expression were closely correlated with the degree of infection.

## 1 INTRODUCTION

Diabetes foot ulcer (DFU) refers to the infection, ulcer, or tissue destruction in the foot of patients with newly diagnosed diabetes or with diabetes history, usually accompanied by lower limb neuropathy or peripheral arterial disease. DFU is an important complication of diabetes, with the prevalence rate of about 2%–10%.^1^ The attack of DFU is acute and severe accompanied by complex clinical manifestations, and there are many kinds of related infectious bacteria. Typical DFU is characterized by rapid progressive necrosis of the severely infected foot. If the patients cannot receive the corresponding treatment timely, the limb and life safety will be greatly threatened.^2^

Surgical removal of necrotic tissue and purulent accumulation is an important treatment for DFU, which plays an important role in promoting the early healing of ulcer surface. According to relevant reports,^3^, ^4^ a series of different but interrelated processes were involved in the healing process of ulcer surface, including coagulation of extracellular matrix, inflammatory reaction, cell migration, proliferation, regeneration, and remodeling. In addition, previous study found^5^ that neutrophils and macrophages could eliminate pathogenic bacteria by inducing autophagy after the pathogenic bacteria invade into the human body, thus promoting tissue healing. However, the relationship between the severity of infection in DFU patients with inflammatory factors and autophagic proteins is still unclear.

Ulcer healing in diabetic foot patients is a complex and dynamic process that involves the collaboration between inflammatory cells and biochemical mediators stimulated by various factors. 152 DFU patients admitted in our hospital during July 2020 to March 2022 were chosen as the research objects in this study, and were further grouped in line with the severity of infection. The expression of inflammatory factors and autophagy‐related proteins in the granulation tissue of DFU patients was examined by automatic bacterial analyzer and immunohistochemistry, and their relationship with infection was analyzed, so as to provide new ideas for the wound repair mechanism of diabetic foot and the treatment of diabetic foot, finally providing some reference for clinical treatment of DFU.

## 2 MATERIALS AND METHODS

### 2.1 General materials

This study was a cross‐sectional study. Based on past experience, the standard deviation was expected to be 30, a two‐sided test was required, the α was 0.05, the allowable error was 5, and the formula was *n* = [(1.96 × 30)/5]^2^. A sample size of 139 was obtained based on the formula, and considering a 10% loss to follow‐up rate, this example would require at least 152 patients to be included as study subjects. A total of 152 DFU patients in our hospital during July 2020 to March 2022 were chosen as the DFU group, including 86 males and 66 females aged between 33 and 80, with an average age of 53.64 ± 8.82. Among the selected subjects, 98 cases were in infection stage and 54 cases were in infection control stage. The patients were further graded as the mild (51 cases), the moderate (65 cases), and the severe infection group (36 cases) according to the Wagner grading criteria.^2^ According to whether the patients had adverse cardiovascular events (angina, acute myocardial infarction, severe arrhythmia, heart failure, and coronary heart disease death), the patients were divided into the occurrence group (48 cases) and the nonoccurrence group (104 cases). Inclusion criteria: (1) All patients met the diagnostic criteria for DFU in the references^6^; (2) Patients who could cooperate with the study to collect the wound tissue; (3) Patients who have not received relevant treatment before admission. Exclusion criteria: (1) Patients with abnormal coagulation function; (2) patients with pneumonia, sepsis, malignant tumors and immune system disorders; (3) patients with inflammatory diseases, including pancreatitis, hepatitis, gastroenteritis, appendicitis, prostatitis, vaginitis, frozen shoulder, otitis media, and thyroiditis were excluded through laboratory testing and preliminary evaluation by doctors after admission; (4) patients with obvious abnormal liver and kidney function; (5) patients with previous cardiovascular events (myocardial infarction and heart failure) and cerebrovascular events (cerebral infarction, cerebral hemorrhage, and brain tumor). As a comparison, 67 patients with foot burns (39 males and 28 females aged between 35 and 80, with an average age of 55.85 ± 7.73) in our hospital during the same period were picked as the control group. There was no statistical difference in age and gender between two groups (*p* > .05). The present study was ratified by the Ethics Committee of Dongzhimen Hospital, Beijing University of Chinese Medicine (Approval number: DZMEC‐KY‐2020‐40‐02) and all the steps were conducted following the medical ethics. The Clinical trial registration number was ChiCTR2000041443.

### 2.2 Methods

The deep tissue samples as an adjunct to swab samples from base of ulcer of the patient was collected, and the distribution of pathogenic bacteria on the ulcer surface was examined with the automatic bacterial analyzer (Beijing Haifuda Technology Co. Ltd., model: DY15‐XY).

The expression of inflammatory factors in ulcer granulation tissue was evaluated by real‐time fluorescence qRT‐PCR (qRT‐PCR), including the procalcitonin (PCT), tumor necrosis factor‐α (TNF‐α) and interleukin‐6 (IL‐6). qRT‐PCR^7^: After the granulation tissue on the ulcer surface is fully split and homogenized, RNA is extracted by Trizol, and the concentration is detected. After centrifuging in a centrifuge for 20 min, the supernatant was collected and was mixed with isopropanol. After standing, the mixture was centrifuged and the supernatant was removed. One milliliter of 75% ethanol was added to the precipitated species, mixed well, and was centrifuged. After the supernatant was removed, the centrifugal tube was stood still and was added with 30 μL RNase‐free dd H_2_O. The centrifugal tube was stood still at room temperature, and the mixture was fully mixed. Wait for the precipitation to be completely dissolve, and the total RNA of the sample is obtained. A small amount of RNA solution was taken for gel electrophoresis. cDNA was obtained through the reverse transcription of RNA. Then, cDNA was used as a template to perform the 20 μL system PCR reaction. The PCR cycle parameters are 95°C for 5 min, 95°C for 15 s, 58°C for 30 s and 72°C for 30 s, with 20 cycles. GAPDH was taken as the internal parameter, and the fold change was calculated by the equation 2^△△*Ct*^.

The content of autophagy‐related proteins (Beclin‐1, microtubule‐binding light chain protein‐3 [LC3]) in the ulcerated granulation tissue was measured by immunohistochemistry (IHC). IHC^8^: The granulation tissue on the ulcer surface was routinely made into paraffin sections, which were routinely dewaxed, and then immersed in 0.01 mol/L citrate buffer (pH6.0) for microwave repair for 20 min. After cooling, the slices were incubated with 3% hydrogen peroxide for 30 min at room temperature for the inactivation of endogenous enzymes, and then were sealed with 5% BSA for 30 min at room temperature. Each slice was incubated with 1:250 diluted antibody at 4°C overnight. After cleaning, the slice was incubated with biotinylated goat anti‐rabbit IgG for 40 min. Then, the slice was incubated with SABC for 30 min at room temperature for staining. The section was finally colored with DAB, and the positive expression was shown as brownish yellow. The slices were washed with PBS (pH 7.4) for three times between steps. After being counterstained with hematoxylin, the dehydration, transparency, and sealing were carried out in turn, the slices were finally observed by optical microscope. With the help of the computer image processing system, 10 visual fields were randomly selected from each slice to measure the average optical density of positive staining cells.

Examination of glycated hemoglobin: After fasting for 8–10 h, venous blood (2 mL) was collected from patients with DFU and put into EDTA‐K2 anticoagulant tube. The level of glycated hemoglobin was measured by high‐performance liquid chromatography using Tosoh G8 Hba1c analyzer and matching reagents (Tosoh Company, Japan).

### 2.3 Statistical analysis

In this study, the enumeration data, such as the distribution of pathogenic bacteria, were shown as (cases [%]). The measurement data such as the inflammatory factors, autophagic protein expression, and so on were tested by normal distribution, which were consistent with normal distribution, and were all expressed in the form of ($\bar{x}\pm s$). The measurement data between two groups were compared by *t*‐test. The multiple groups were analyzed by one‐way analysis of variance (ANOVA), and the LSD‐*t*‐test was used for post hoc analysis. Pearson was used to analyze the correlation between glycated hemoglobin and the expression of inflammatory factors and autophagy proteins in patients with DFU. SPSS23.0 software was used for statistical data analysis, and *p* < .05 was regarded as statistically significant difference.

## 3 RESULTS

### 3.1 Distribution of pathogenic bacteria on ulcer surface in DFU patients

The surface infection of DFU patients is mostly caused by gram‐negative and gram‐positive bacteria, with *Pseudomonas aeruginosa* predominating among the gram‐negative bacteria and *Staphylococcus aureus* among the gram‐positive bacteria (Table 1).

**Table 1:** Distribution of pathogenic bacteria on surface in diabetic foot ulcer (DFU) patients (cases [%]).

Groups	DFU group (*n* = 152)		Control group (*n* = 67)	
	Number of bacterial strains	Proportion (%)	Number of bacterial strains	Proportion (%)
Gram‐negative bacteria	58	38.15	27	40.30
*Escherichia coli*	6	10.34	3	11.11
*Klebsiella pneumoniae*	7	12.07	4	14.81
*Enterobacter cloacae*	5	8.62	2	7.41
*Pseudomonas aeruginosa*	25	43.10	11	40.74
*Proteus*	11	18.97	5	18.52
Other	4	6.90	2	7.41
Gram‐positive bacteria	87	57.24	36	53.73
*Enterococcus faecalis*	12	13.79	6	16.67
*Staphylococcus superficialis*	21	24.14	5	13.89
*Streptococcus*	11	12.64	2	5.56
*Staphylococcus aureus*	30	34.48	15	41.67
Other	13	14.94	8	22.22
Fungus	7	4.61	4	5.97
*candida albicans*	4	57.14	3	75.00
Other	3	42.86	1	25.00

### 3.2 Expression of inflammatory factors in ulcer granulation tissue

Compared with the control group, the contents of PCT, TNF‐α, and IL‐6 were higher was lower in the DFU infection control group without significant difference (*p* > .05). The content of PCT, TNF‐α, and IL‐6 was markedly elevated in infection stage group than the control group (*p* < .05). The infection stage group had much higher content of PCT, TNF‐α, and IL‐6 than the infection control group (*p* < .001, Table 2).

**Table 2:** Expression of inflammatory factors in ulcer granulation tissue ($\bar{x}\pm s$).

Groups	Cases	Procalcitonin (ng/L)	Tumor necrosis factor‐α (ng/L)	Interleukin‐6 (ng/L)
Control group	67	3.14 ± 1.74	1.85 ± 0.86	1.73 ± 0.80
Diabetic foot ulcer group				
Infection control group	54	3.87 ± 1.23^a^	2.24 ± 1.19^a^	2.09 ± 1.32^a^
Infection stage group	98	6.19 ± 2.24^a^, ^b^	4.58 ± 1.29^a^, ^b^	3.62 ± 1.87^a^, ^b^
*F*		58.85	135.63	37.80
*p*		<.001	<.001	<.001

^a^ *p* < .05 compared with the control group.

^b^ *p* < .05 compared with the infection control group.

### 3.3 Expression of autophagy protein in ulcer granulation tissue

In comparison with the control group, the expression of Beclin‐1 and LC3 in the infection control group were slightly lower (*p* > .05), while the levels of PCT, TNF‐α, and IL‐6 in the infection stage group were significantly higher (*p* < .05, Table 3). Compared with the infection control group, the expression of Beclin‐1 and LC3 in the infection stage group were sharply lower (*p* < .001, Table 3).

**Table 3:** Expression of autophagy protein in ulcer granulation tissue ($\bar{x}\pm s$).

Groups	Cases	Beclin‐1	LC3
Control group	67	0.92 ± 0.25	1.47 ± 0.55
Diabetic foot ulcer group			
Infection control group	54	0.87 ± 0.24^a^	1.36 ± 0.42^a^
Infection stage group	98	0.58 ± 0.04^a^, ^b^	0.65 ± 0.32^a^, ^b^
*F*		81.58	89.55
*p*		<.001	<.001

^a^ *p* < .05 compared with the control group.

^b^ *p* < .05 compared with the infection control group.

### 3.4 Expression of inflammatory factors in ulcer granulation tissue of patients with different degrees of infection

In comparison with the mild group, the contents of PCT, TNF‐α, and IL‐6 were markedly higher in the moderate group (*p* < .05). PCT, TNF‐α, and IL‐6 levels were memorably higher and Beclin‐1 and LC3 levels were prominently lower in the severe group than the moderate group (*p* < .001, Table 4).

**Table 4:** Expression of inflammatory factors in ulcer granulation tissue of patients with different degrees of infection ($\bar{x}\pm s$).

Groups	Cases	Procalcitonin (ng/L)	Tumor necrosis factor‐α (ng/L)	Interleukin‐6 (ng/L)
Mild group	51	4.85 ± 3.07	3.49 ± 1.55	3.12 ± 1.33
Moderate group	65	6.69 ± 3.38^a^	4.68 ± 1.37^a^	4.10 ± 1.58^a^
Severe group	36	8.26 ± 4.20^a^, ^b^	5.64 ± 1.78^a^, ^b^	5.64 ± 1.87^a^, ^b^
*F*		10.33	21.41	26.97
*p*		<.001	<.001	<.001

^a^ *p* < .05 compared with the mild group.

^b^ *p* < .05 compared with the moderate group.

### 3.5 Expression of inflammatory factors in DFU patients with or without cardiovascular events

The expression levels of PCT, TNF‐α and IL‐6 in the occurrence group were significantly higher than these in the nonoccurrence group (*p* < .001, Table 5).

**Table 5:** Expression of inflammatory factors in diabetic foot ulcer patients with or without cardiovascular events ($\bar{x}\pm s$).

Groups	Cases	Procalcitonin (ng/L)	Tumor necrosis factor‐α (ng/L)	Interleukin‐6 (ng/L)
The occurrence group	48	9.36 ± 3.45	6.60 ± 1.92	6.42 ± 1.69
The nonoccurrence group	104	4.67 ± 1.09	3.87 ± 1.62	3.48 ± 1.60
*t*		12.607	9.098	10.345
*p*		<.001	<.001	<.001

### 3.6 Expression of autophagic proteins in ulcer granulation tissue of patients with different degrees of infection

The expression level of Beclin‐1 and LC3 was sharply lower in the moderate group than the mild group (*p* < .05). The Beclin‐1 and LC3 were lowly expressed in the severe group than the moderate group (*p* < .001, Table 6).

**Table 6:** Expression of autophagic proteins in ulcer granulation tissue of patients with different degrees of infection ($\bar{x}\pm s$).

Groups	Cases	Beclin‐1	LC3
Mild group	51	0.73 ± 0.14	0.62 ± 0.14
Moderate group	65	0.62 ± 0.09^a^	0.54 ± 0.11^a^
Severe group	36	0.41 ± 0.11^a^, ^b^	0.29 ± 0.12^a^, ^b^
*F*		84.49	79.74
*p*		<.001	<.001

^a^ *p* < .05 compared with the mild group.

^b^ *p* < .05 compared with the moderate group.

### 3.7 Correlation between glycated hemoglobin and the expression of inflammatory factors and autophagy proteins in patients with DFU

Compared with the mild group, the level of glycated hemoglobin in the moderate group was significantly increased (*p* < .05). Besides, the level of glycated hemoglobin in the severe group was elevated compared with the moderate group (*p* < .001, Table 7). Pearson correlation analysis showed that glycated hemoglobin in patients with DFU was positively correlated with PCT, TNF‐α and IL‐6 levels (*p* < .05), and negatively correlated with Beclin‐1 and LC3 levels (*p* < .001, Table 8).

**Table 7:** Glycated hemoglobin levels in diabetic foot ulcer patients with different conditions.

Groups	Cases	Glycated hemoglobin (%)
Mild group	51	5.28 ± 2.62
Moderate group	65	7.33 ± 2.40^a^
Severe group	36	9.12 ± 2.74^a^, ^b^
*F*		24.39
*p*		<.001

^a^ *p* < .05 compared with the mild group.

^b^ *p* < .05 compared with the moderate group.

**Table 8:** Correlation between glycated hemoglobin and the expression of inflammatory factors and autophagy proteins in patients with diabetic foot ulcer.

Indicators	Glycated hemoglobin	
	*r*	*p*
Procalcitonin	.278	.001
Tumor necrosis factor‐α	.307	<.001
Interleukin‐6	.234	.004
Beclin‐1	−.320	<.001
LC3	−.371	<.001

## 4 DISCUSSION

In recent years, with the increasing incidence rate of diabetes, the corresponding incidence rate of complications has also increased. DFU is a common complication of diabetes, which is mainly induced by hyperglycemia, causing peripheral nerve and vascular diseases, drying and inflammation of the foot skin, and finally forming ulcers, which will not heal for a long time, reducing the life quality. In addition, diabetes will greatly reduce the immune function of the body and increase the probability of infection. The occurrence of infection can further aggravate the patient's condition, thus forming a vicious circle.^9^ In this study, it was found that *P. aeruginosa* and *S. aureus* were the main infectious bacteria in DFU surface. Wound debridement, reduction of ulcer load, drug treatment, wound dressing, and prevention of infection by keeping the ulcer clean are the gold standards for treating DFU.^10^ Despite advances in wound healing methods, chronic DFU are still a key clinical problem associated with expensive and long‐term treatment, amputation risk, and high incidence rate and mortality. The healing of ulcer surface is a complex physiological process, including four stages: steady state/coagulation, inflammatory cell recruitment, proliferation, and maturation. Angiogenesis and granulation tissue formation play a key role in wound healing by providing nutrition, oxygen, and matrix. Damaged granulation tissue formation and angiogenesis are prominent features of foot ulcers in incorrigible DFU patients.

It is believed^11^ that in diabetes patients with hyperglycemia, abnormal proliferation of peripheral nerve and vascular cells occurs, and inflammatory reaction may be the main reason why DFU is difficult to cure. Long‐term hyperglycemia in diabetic patients can lead to narrowing or even obstruction of the lumen of peripheral blood vessels, resulting in vascular endothelial cell damage, with a large number of red blood cell aggregation, platelet adhesion, and microvascular embolism at the injured site, and eventually acral ulcers.^12^ Due to lipid metabolism disorders and internal constipation disorders, the characteristics of the tubes and blood are changed, which cause the increase of fibrin, the aggregation of red blood cells, the adhesion of platelets, and the formation of adherent thrombosis to result in acral ischemia and hypoxia, thus leading to ulcer formation. It has been reported that cytokines released from adipose tissue may be involved in initiating and promoting pro‐inflammatory states, and contribute to the regulation of insulin resistance and insulin sensitivity and secretion.^13^ In this study, by analyzing the level of inflammatory factors in the ulcer granulation tissue, we found that the content of inflammatory factors such as PCT, TNF‐α, and IL‐6 largely increased with the progress of the disease and the aggravation of infection, suggesting that inflammatory reaction may participate in the progression of DFU. Previous studies^14^ have shown that the level of inflammatory factors in diabetic patients is in a state of chronic hypoinflammation, and the level of inflammatory factors is closely related to the occurrence and progression of diabetes and related complications. Moreover, the study has shown^15^ that IL‐6 levels are positively correlated with the condition of patients with type 2 diabetes. High IL‐6 levels can lead to weakened tissue repair ability, and excessive release of TNF‐α can induce pathorational damage and disrupt the body's immune balance. PCT is a protein precursor of calcitonin hormone, which is synthesized and secreted by C cells in the thyroid gland. It has been suggested that post‐inflammatory PCT is produced by liver and peripheral blood mononuclear cells and is regulated by lipopolysaccharides and sepsis‐associated cytokines.^16^ It has also been reported that PCT is a more accurate marker for differential diagnosis of bacterial infection compared with CRP.^17^ PCT has been proved to play an important role in the diagnosis of DFU ulcer.^18^ However, serum PCT levels were primarily evaluated in the treatment and follow‐up of infectious ulcers, and the results showed no role in differentiating mild‐to‐moderate and severe infections of diabetic ulcers.^18^ However, it has also been suggested that,^19^ the optimal cut‐off concentration of PCT is 0.25 ng/mL with the sensitivity and specificity are 63.6% and 83.2%, is considered to be a valuable biomarker for diagnosing infection, which serves to be of little value for the early diagnosis of diabetic foot ulcers due to its low sensitivity. Foreign study^20^ has shown that abdominal fat cells in diabetic patients can secrete a large amount of TNF‐α, and TNF‐α enters other cells through paracrine or endocrine, interfering with insulin signaling and inhibiting the uptake of glucose by somatic cells, finally resulting in diabetes. In addition, in foot infection, the immune system activates monocyte activity, secretes and releases excessive TNF‐α. TNF‐α can cause pathological damage, damage vascular endothelial cells, aggravate foot disease, and TNF‐α has a certain correlation with atherosclerosis.^21^ Research showed that,^15^ the content of TNF‐α increased in the healing process of chronic wound of infected DFU, indicating that TNF‐α was an effective pro‐inflammatory cytokine and was closely related to the progression of DFU. In addition, IL‐6 is one of the detectable inflammatory cytokines in early serum and plays an important role in the process of bacterial infection, especially in the induction of CRP and fibrinogen synthesis in the liver.^22^ As a result, it has been suggested that IL‐6 cytokines may increase earlier than CRP during bacterial infections and enable early diagnosis.^23^ However, there are currently limited studies evaluating the role of serum IL‐6 levels in diabetic ulcers. It is reported^24^ that IL‐6 stimulates the inflammatory and autoimmune processes of many diseases, such as diabetes, atherosclerosis, depression, Alzheimer's disease, systemic lupus erythematosus, multiple myeloma, prostate cancer, Behcet's disease, and rheumatoid arthritis. IL‐6 has certain value in the course monitoring of type 2 diabetes patients with foot infection.^25^ Therefore, these above research results suggested that TNF‐ α, PCT, and IL‐6 could be used as diagnostic factors of DFU. In addition, the results of this study showed that the levels of PCT, TNF‐α, and IL‐6 in the DFU patients with cardiovascular events group were significantly higher than those in the nonoccurrence group, suggesting that abnormal inflammatory response in DFU patients could promote the occurrence of adverse cardiovascular events. Poor blood glucose control in patients with DFU is one of the important causes of stroke. Hyperglycemia can lead to dysfunction of vascular endothelial cells and increase vascular wall permeability, thus accelerating the process of atherosclerosis.^26^ At the same time, hyperglycemia can also promote platelet aggregation, form thrombosis, and block cerebral blood vessels, leading to the occurrence of stroke. Increased inflammation may be an important factor in the increased brain damage after diabetic foot stroke, and oxygen and nitrogen free radicals released by macrophages and neutrophils that are extremely toxic to neurons.^27^ The study^28^ has confirmed that acute inflammatory responses in the post‐stroke brain and macrophages after LPS stimulation are disturbed under diabetic conditions, and these changes are associated with increased stroke‐induced damage. Overall, the data suggest^29^ that the early inflammatory response in the diabetic brain is poorly controlled and is associated with an exacerbation of stroke‐induced damage.

As the first line of defense of the body and immunity, skin includes several important types of immune active cells. Among them, macrophages are the main cell subgroup in the dermis and the first line of defense against pathogens. When the pathogen enters the damaged skin and causes infection, macrophages can migrate to the infected site to remove the pathogen through allophagocytosis and phagocytosis.^30^ Autophagy is an intracellular process that plays a vital role in the host's defense against invasive pathogens. The damage of autophagy further promotes β cell dysfunction and the progression of diabetes. The results of this study showed that the content of Beclin‐1 and LC3 largely decreased in DFU patients, and gradually decreased with the aggravation of infection. Autophagy may play an important role in the development of DFU. The above studies suggested that diabetes might be significantly related to autophagy. It was similar to the results of related studies. Animal studies have proved^31^ that high glucose can inhibit cell viability, increase cell apoptosis, accompanied by suppressed LC‐3 and strengthened P62. In addition, the levels of Beclin‐1 and LC3 in ulcer granulation tissue of infected DFU patients were strongly reduced.^9^ The above results indicated that high glucose inhibited autophagy, and the expression of autophagy protein in granulation tissue of DFU patients was closely related to infection. Glycated hemoglobin is an important indicator for the diagnosis and monitoring of diabetes, which can reflect the average blood glucose level in the past 2–3 months.^32^ In recent years, studies have found that glycated hemoglobin is closely related to the occurrence and development of DFU, and the increase of glycated hemoglobin level can lead to the aggravation of lower limb vascular lesions and neuropathy, thereby increasing the risk of DFU.^33^, ^34^ Pearson correlation analysis showed that glycated hemoglobin in patients with DFU was positively correlated with PCT, TNF‐α and IL‐6 levels, and negatively correlated with Beclin‐1 and LC3 levels. These results suggested that elevated glycated hemoglobin might promote the occurrence and development of DFU by enhancing the expression of inflammatory factors and inhibiting the expression of autophagy proteins.

In general, *P. aeruginosa* and *S. aureus* were predominant bacterial in DFU infections. The expression of inflammatory factor was obviously elevated and autophagy protein expression was strongly decreased in ulcer granulation tissue of DFU patients, which were closely correlated with the degree of infection. It is necessary to detect the disease early, diagnose it early, control the occurrence of infection, properly guide the patient's autophagy, help the patient recover, and accumulate experience and methods for the treatment of diabetic foot infection for the majority of medical staff. However, this study still has certain limitations. Limited sample size and unknown duration of ulcer and infection are one of the limitations of this study, which needed be further verified in following study. Correlation between the expression of inflammatory factors and autophagy proteins and infection in ulcer granulation tissue of diabetic foot patients is needed.

## AUTHOR CONTRIBUTIONS

**Lijuan Deng**: Data curation; formal analysis; investigation; methodology; resources; software; supervision; validation; visualization; writing—original draft; writing—review and editing. **Gang Wang**: Data curation; formal analysis; investigation; methodology; resources; software; supervision; validation; visualization; writing—original draft; writing—review and editing. **Shang Ju**: Conceptualization; data curation; formal analysis; funding acquisition; investigation; methodology; project administration; resources; software; supervision; validation; visualization; writing—review and editing.

## CONFLICT OF INTEREST STATEMENT

The authors declare no conflict of interest.

## Data Availability

The datasets used and/or analyzed during the current study are available from the corresponding author upon reasonable request.

## References

[1] Riedel U , Schüßler E , Härtel D , Keiler A , Nestoris S , Stege H . Wundbehandlung bei diabetes und diabetischem fußulkus. Der Hautarzt. 2020;71(11):835‐842. 10.1007/s00105-020-04699-9 33044558

[2] Carro GV , Saurral R , Witman EL , et al. Diabetic foot attack. Pathophysiological description, clinical presentation, treatment and outcomes. Medicina. 2020;80(5):523‐530. 33048798

[3] Rodrigues M , Kosaric N , Bonham CA , Gurtner GC . Wound healing: a cellular perspective. Physiol Rev. 2019;99(1):665‐706. 30475656 10.1152/physrev.00067.2017 PMC6442927

[4] Raziyeva K , Kim Y , Zharkinbekov Z , Kassymbek K , Jimi S , Saparov A . Immunology of acute and chronic wound healing. Biomolecules. 2021;11(5):700. 34066746 10.3390/biom11050700 PMC8150999

[5] Yao RQ , Ren C , Xia ZF , Yao YM . Organelle‐specific autophagy in inflammatory diseases: a potential therapeutic target underlying the quality control of multiple organelles. Autophagy. 2021;17(2):385‐401. 32048886 10.1080/15548627.2020.1725377 PMC8007140

[6] Schaper NC , van Netten JJ , Apelqvist J , Bus SA , Hinchliffe RJ , Lipsky BA , IWGDF Editorial Board . Practical guidelines on the prevention and management of diabetic foot disease (IWGDF 2019 update). Diabetes Metab Res Rev. 2020;36(suppl 1):e3266. 32176447 10.1002/dmrr.3266

[7] Bustin SA , Nolan T . RT‐qPCR testing of SARS‐CoV‐2: a primer. Int J Mol Sci. 2020;21(8):3004. 32344568 10.3390/ijms21083004 PMC7215906

[8] Ieni A , Cardia R , Giuffrè G , Rigoli L , Caruso RA , Tuccari G . Immunohistochemical expression of autophagy‐related proteins in advanced tubular gastric adenocarcinomas and its implications. Cancers. 2019;11(3):389. 30893939 10.3390/cancers11030389 PMC6468613

[9] Aitcheson SM , Frentiu FD , Hurn SE , Edwards K , Murray RZ . Skin wound healing: normal macrophage function and macrophage dysfunction in diabetic wounds. Molecules. 2021;26(16):4917. 34443506 10.3390/molecules26164917 PMC8398285

[10] Rai V , Moellmer R , Agrawal DK . Stem cells and angiogenesis: implications and limitations in enhancing chronic diabetic foot ulcer healing. Cells. 2022;11(15):2287. 35892584 10.3390/cells11152287 PMC9330772

[11] Liu Z , Dumville JC , Hinchliffe RJ , et al. Negative pressure wound therapy for treating foot wounds in people with diabetes mellitus. Cochr Database System Rev. 2018;10(10):010318. 10.1002/14651858.CD010318.pub3 PMC6517143 30328611

[12] Del Cuore A , Pipitone RM , Casuccio A , et al. Metabolic memory in diabetic foot syndrome (DFS): MICRO‐RNAS, single nucleotide polymorphisms (SNPs) frequency and their relationship with indices of endothelial function and adipo‐inflammatory dysfunction. Cardiovasc Diabetol. 2023;22(1):148. 37365645 10.1186/s12933-023-01880-x PMC10294440

[13] Tuttolomondo A , Di Raimondo D , Casuccio A , et al. Endothelial function, adipokine serum levels, and white matter hyperintesities in subjects with diabetic foot syndrome. J Clin Endocrinol Metab. 2019;104(9):3920‐3930. 30977833 10.1210/jc.2018-02507

[14] Dörr S , Freier F , Schlecht M , Lobmann R . Bacterial diversity and inflammatory response at first‐time visit in younger and older individuals with diabetic foot infection (DFI). Acta Diabetol. 2021;58(2):181‐189. 32944830 10.1007/s00592-020-01587-5

[15] Dhamodharan U , Teena R , Vimal Kumar R , Changam SS , Ramkumar KM , Rajesh K . Circulatory levels of B‐cell activating factor of the TNF family in patients with diabetic foot ulcer: association with disease progression. Wound Repair Regen. 2019;27(5):442‐449. 31041853 10.1111/wrr.12720

[16] Liang P , Yu F . Value of CRP, PCT, and NLR in prediction of severity and prognosis of patients with bloodstream infections and sepsis. Front Surg. 2022;9:857218. 35345421 10.3389/fsurg.2022.857218 PMC8957078

[17] Tian T , Wei B , Wang J . Study of c‐reactive protein, procalcitonin, and immunocyte ratios in 194 patients with sepsis. BMC Emerg Med. 2021;21(1):81. 34233608 10.1186/s12873-021-00477-5 PMC8265098

[18] Hadavand F , Amouzegar A , Amid H . Pro‐calcitonin, erythrocyte sedimentation rate and C‐reactive protein in predicting diabetic foot ulcer characteristics; a cross sectional study. Arch Acad Emerg Medicine. 2019;7(1):37. PMC6732205 31555767

[19] Omar J , Ahmad N , Che‐Soh N , et al. Serum procalcitonin (PCT) ‐ is there a role as an early biomarker in infected diabetic foot ulcer (IDFU) patients? Malays Orthop J. 2023;17(2):62‐69. 37583519 10.5704/MOJ.2307.010 PMC10425005

[20] Otoda T , Sekine A , Uemoto R , et al. Albuminuria and serum tumor necrosis factor receptor levels in patients with type 2 diabetes on SGLT2 inhibitors: a prospective study. Diabetes Ther. 2024;15(1):127‐143. 37883001 10.1007/s13300-023-01488-0 PMC10786751

[21] Galstyan KO , Nedosugova LV , Martirosian NS , et al. Modification of tumor necrosis Factor‐α and C‐C motif chemokine ligand 18 secretion by monocytes derived from patients with diabetic foot syndrome. Biology. 2019;9(1):3. 31877847 10.3390/biology9010003 PMC7168305

[22] Gür A , Oguzturk H , Köse A , et al. Prognostic value of procalcitonin, CRP, serum amyloid A, lactate and IL‐6 markers in liver transplant patients admitted to ED with suspected infection. In Vivo. 2017;31(6):1179‐1185. 29102943 10.21873/invivo.11187 PMC5756649

[23] Zhu S , Zeng C , Zou Y , Hu Y , Tang C , Liu C . The clinical diagnostic values of SAA, PCT, CRP, and IL‐6 in children with bacterial, viral, or co‐infections. Int J Gen Med. 2021;14:7107‐7113. 34729020 10.2147/IJGM.S327958 PMC8554317

[24] Balasubramanian GV , Chockalingam N , Naemi R . The role of cutaneous microcirculatory responses in tissue injury, inflammation and repair at the foot in diabetes. Front Bioeng Biotechnol. 2021;9:732753. 34595160 10.3389/fbioe.2021.732753 PMC8476833

[25] Dmitriyeva M , Kozhakhmetova Z , Urazova S , Kozhakhmetov S , Turebayev D , Toleubayev M . Inflammatory biomarkers as predictors of infected diabetic foot ulcer. Curr Diabetes Rev. 2022;18(6):e280921196867. 34602039 10.2174/1573399817666210928144706

[26] Shukla V , Shakya AK , Perez‐Pinzon MA , Dave KR . Cerebral ischemic damage in diabetes: an inflammatory perspective. J Neuroinflammation. 2017;14(1):21. 28115020 10.1186/s12974-016-0774-5 PMC5260103

[27] Jackson L , Dumanli S , Johnson MH , Fagan SC , Ergul A . Microglia knockdown reduces inflammation and preserves cognition in diabetic animals after experimental stroke. J Neuroinflammation. 2020;17(1):137. 32345303 10.1186/s12974-020-01815-3 PMC7189436

[28] Hong P , Li FX , Gu RN , et al. Inhibition of NLRP3 inflammasome ameliorates cerebral ischemia‐reperfusion injury in diabetic mice. Neural Plast. 2018;2018:1‐8. 10.1155/2018/9163521 PMC5941718 29853850

[29] Nedosugova LV , Markina YV , Bochkareva LA , et al. Inflammatory mechanisms of diabetes and its vascular complications. Biomedicines. 2022;10(5):1168. 35625904 10.3390/biomedicines10051168 PMC9138517

[30] Xie X , Zhong R , Luo L , et al. The infection characteristics and autophagy defect of dermal macrophages in STZ‐induced diabetic rats skin wound *Staphylococcus aureus* infection model. Immun Inflamm Dis. 2021;9(4):1428‐1438. 34647429 10.1002/iid3.492 PMC8589369

[31] Song FC , Yuan JQ , Zhu MD , et al. High glucose represses the proliferation of tendon fibroblasts by inhibiting autophagy activation in tendon injury. Biosci Rep. 2022;42(3):BSR20210640. 35293974 10.1042/BSR20210640 PMC8935382

[32] Hsia DS , Rasouli N , Pittas AG , et al. D2d Research Group Implications of the hemoglobin glycation index on the diagnosis of prediabetes and diabetes. J Clin Endocrinol Metab. 2020;105(3):e130‐e138. 31965161 10.1210/clinem/dgaa029 PMC7015453

[33] Casadei G , Filippini M , Brognara L . Glycated hemoglobin (HbA1c) as a biomarker for diabetic foot peripheral neuropathy. Diseases. 2021;9(1):16. 33671807 10.3390/diseases9010016 PMC8006047

[34] Maiya AG , Parameshwar A , Hande M , Nandalike V . Relationship between glycated hemoglobin and vibration perception threshold in diabetic peripheral neuropathy. Int J Low Extrem Wounds. 2020;19(2):120‐124. 31838926 10.1177/1534734619882173

